# Evaluation of Abdominal CT Obtained Using a Deep Learning-Based Image Reconstruction Engine Compared with CT Using Adaptive Statistical Iterative Reconstruction

**DOI:** 10.5334/jbsr.2638

**Published:** 2022-04-08

**Authors:** Yeo Jin Yoo, In Young Choi, Suk Keu Yeom, Sang Hoon Cha, Yunsub Jung, Hyun Jong Han, Euddeum Shim

**Affiliations:** 1 Korea University Ansan Hospital, KR; 2 Korea University Medical Center, KR; 3 GE Healthcare, KR

**Keywords:** deep learning-based image reconstruction, computed tomography, image quality

## Abstract

**Purpose:**

To compare the image quality of CT obtained using a deep learning-based image reconstruction (DLIR) engine with images with adaptive statistical iterative reconstruction-V (AV).

**Materials and Methods:**

Using a phantom, the noise power spectrum (NPS) and task-based transfer function (TTF) were measured in images with different reconstructions (filtered back projection [FBP], AV30, 50, 100, DLIR-L, M, H) at multiple doses. One hundred and twenty abdominal CTs with 30% dose reduction were processed using AV30, AV50, DLIR-L, M, H. Objective and subjective analyses were performed.

**Results:**

The NPS peak of DLIR was lower than that of AV30 or AV50. Compared with AV30, the NPS average spatial frequencies were higher with DLIR-L or DLIR-M. For lower contrast objects, TTF in images with DLIR were higher than those with AV. The standard deviation in DLIR-H and DLIR-M was significantly lower than AV30 and AV50. The overall image quality was the best for DLIR-M (*p < 0.001*).

**Conclusions:**

DLIR showed improved image quality and decreased noise under a decreased radiation dose.

## Introduction

Iterative reconstruction (IR) was developed to decrease image noise [1,2,3]. However, conventional IR has two major limitations: a long reconstruction time and an unnatural image texture [4,5,6]. Adaptive statistical iterative reconstruction V (AV) demonstrates a short reconstruction time [7,8,9]. However, AV has a trade-off between image noise and texture [10].

Recently, image denoising algorithms using artificial neural networks, termed deep learning-based denoising algorithms (DLA), have been developed to overcome the drawbacks of IR [11,12]. Shin et al. showed that although their DLAs achieved less noise than filtered back projection (FBP) and advanced modeled iterative reconstruction (ADMIRE) in low-dose CT, they did not maintain spatial resolution [13]. Jensen et al. reported that TrueFidelity, a type of DLA, improves image quality through noise reduction and increased contrast-to-noise ratio (CNR) in routine-dose CT [14].

Therefore, this study aimed to assess the quality, including noise and spatial resolution, of phantom and abdominal CT with decreased radiation dose using a deep learning-based image reconstruction (DLIR) engine (TrueFidelity, GE Healthcare) with CT using AV, commonly used in abdominal CT.

## Materials and Methods

### Phantom studies

**Figure 1:**
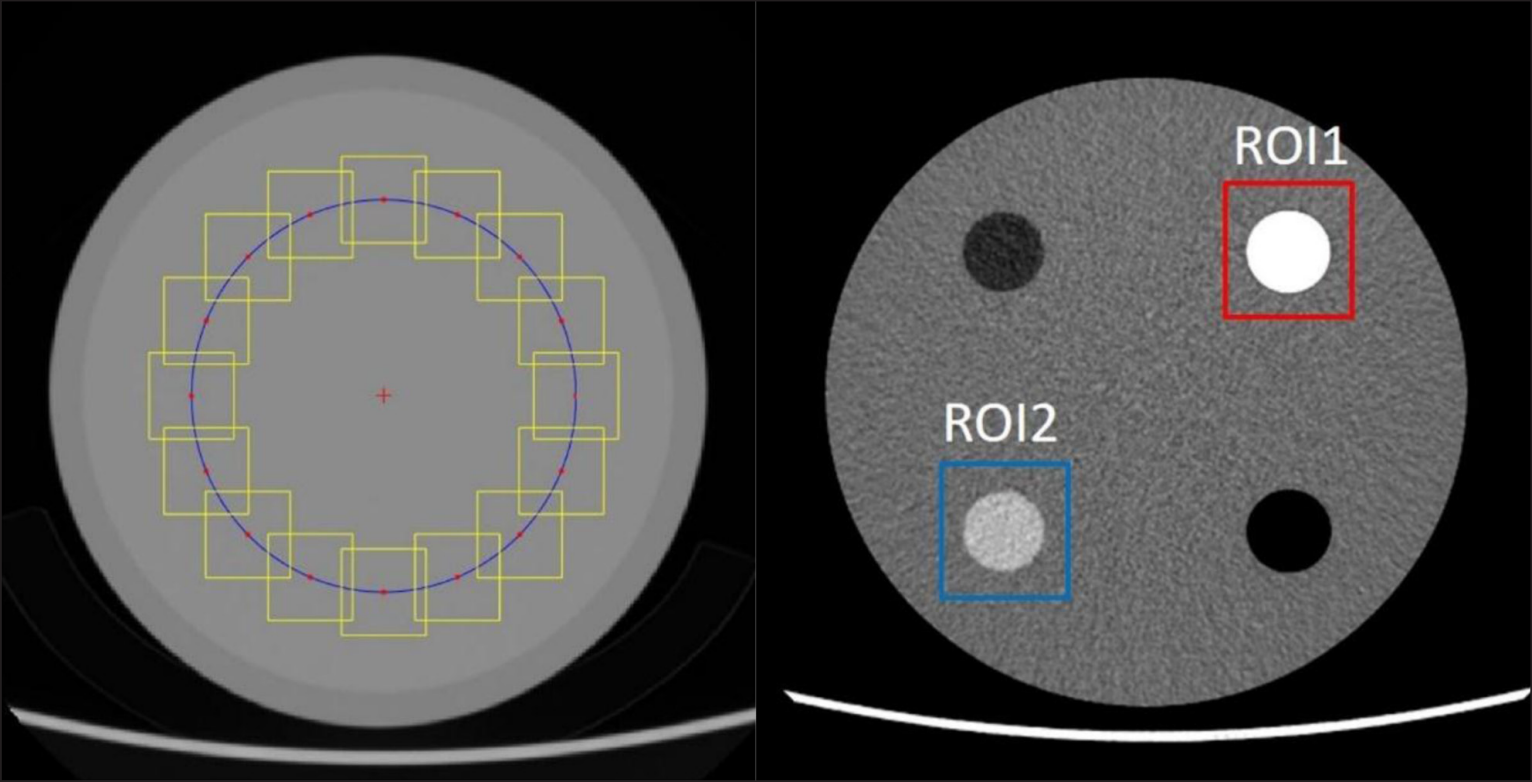
This figure shows (a) noise power spectrum (NPS) and (b) task-based transfer function (TTF) measurement. **(a)** The red cross represents the center of the phantom section, and the blue circle represents the same distance from the center (red cross). The yellow square represents a voxel of interest (25.78 × 25.78 × 12.50 mm) measuring NPS. **(b)** TTF was measured in the region of interest (ROI)1 (bone, 955 HU) and ROI 2 (acrylic, 120 HU) cylinder robs.

The raw data were reconstructed in seven different axial images: FBP and ASIR-V with blending factors of 30%, 50%, or 100% (AV30, AV50, and AV100, respectively). The noise power spectrum (NPS), calculated by the standard Fourier transform technique, determined the amount of noise (magnitude) and noise characteristics (texture) in the spatial frequency domain [15,16,17]. To measure the NPS, we calculated the peak average spatial frequency of module 3 of the American College of Radiology (ACR) phantom (Gammex 464, Sun Nuclear, Middleton, WI, USA) at multiple doses (***Figure 1***). Computed tomography (CT) was performed using following parameters: peak kilovoltage (kVp), 100; beam collimation, 0.625 × 64mm; tube current modulation range 50–250 mAs. The task-based transfer function (TTF) is a representative metric of spatial resolution [13]. We measured TTF in two materials (bone and acrylic) in module 1. To quantify TTF, the spatial frequency (TTF_50%_) was calculated at the point where the Y-axis value became 0.5 in the measured TTF curve. The NPS was implemented and calculated using MATLAB (Version R2017a, The MathWorks, Inc., Natick, MA, USA), and the TTF used imQuest (Duke University) software implemented in MATLAB.

### Patient Studies

This retrospective study was approved by the Institutional Review Board. Two hundred and three patients had undergone abdominal CT (Revolution CT; GE Healthcare) from February 2020 to April 2020. CT scans with 70 different combination of reconstructions, eight large hepatic lesions > 2 cm, and five poor image quality were excluded. The CT of 120 individuals were retrospectively reviewed (***Table 1***). The mean body mass index of patients in this study was 23.6 ± 3.6 (SD).

**Table 1:** Baseline Characteristics of study population.


Demographics	

Age (years)	54.4 ± 20.6

Body mass index	23.1 ± 3.6

Radiation dose	

CTDIvol (mGy)	5.06 ± 1.85

DLP (mGycm)	281.29 ± 92.69


Data are presented as mean±standard deviation. CTDIvol, volume CT dose index; DLP, dose-length product.

### CT Examination and Postprocessing

All patients underwent abdominal CT using a CT system (Revolution, GE Healthcare) that could reconstruct both the AV and DLIR engines. CT was performed using the following parameters: peak kilovoltage (kVp), 100; beam collimation, 0.625 × 128 mm; tube current modulation range 100–550 mAs; noise index, 17; gantry rotation time, 0.6 s; coverage speed, 132.29 mm/s; pitch, 0.992:1; and slice thickness, 2.5 mm. The mean volume CT dose index was 5.06 ± 1.85 (SD) mGy, and the mean dose length product (DLP) was 281.29 ± 92.69 (SD) mGy.cm. A nonioninated contrast medium (Ioversol 320 mg/mL; 2 mL/kg body weight) was administered for contrast enhancement. The timing of the portal venous phase scan was a fixed time-delay technique of 90 s after contrast administration. The raw data were reconstructed in six different reconstructions: FBP, AV30, AV50, and DLIR (DLIR-Low, DLIR-Medium, and DLIR-High).

### Quantitative Analysis

**Figure 2:**
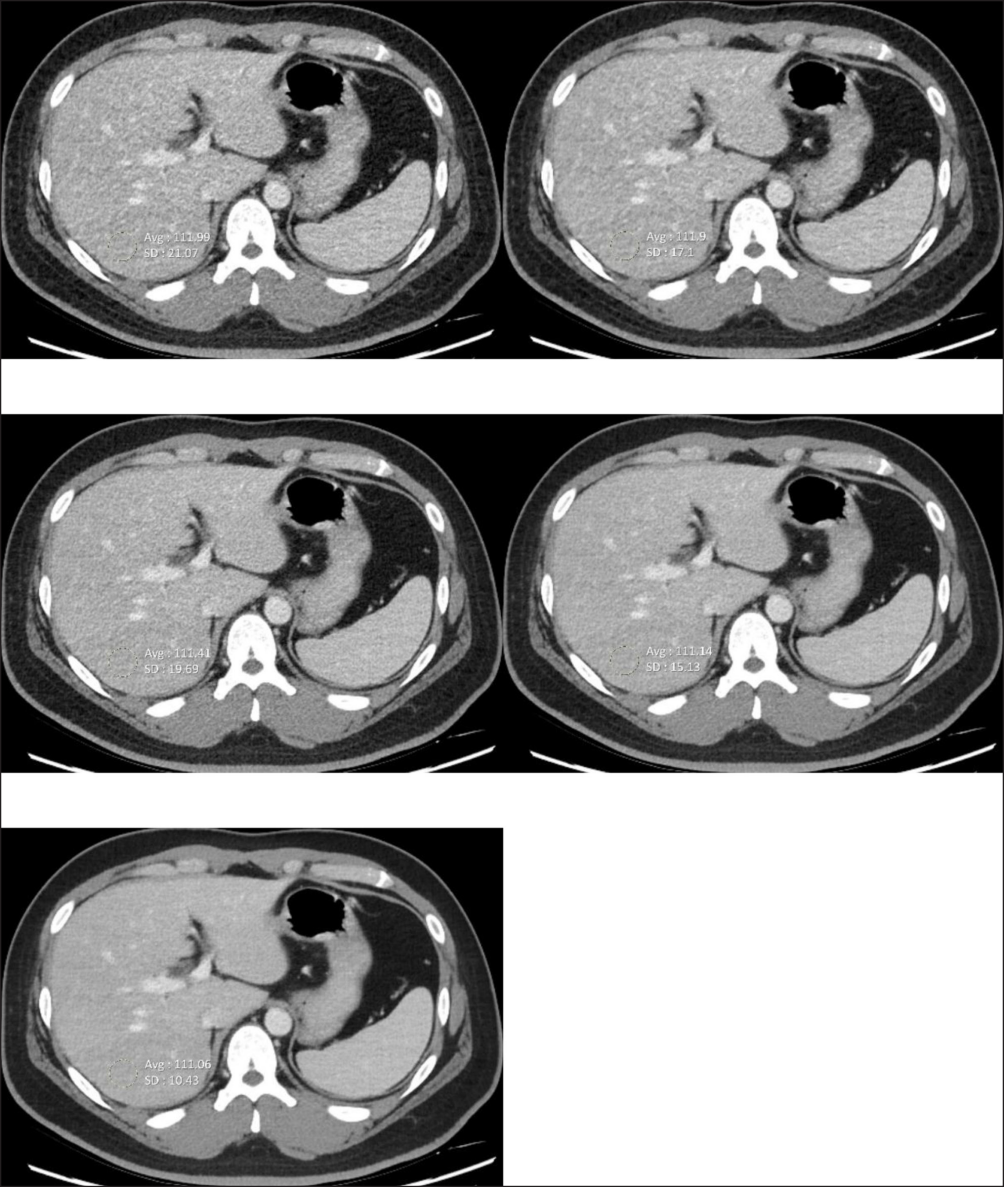
CT images for quantitative analysis of liver **(a)** AV 30 **(b)** AV 50 **(c)** DLIR-L **(d)** DLIR-M **(e)** DLIR-H. The body mass index of this patient is 34.3. FBP, filtered back projection; AV, adaptive statistical iterative reconstruction; DLIR, deep learning-based image reconstruction; DLIR-L, DLIR images with low levels; DLIR-M, DLIR with medium levels.

One radiologist placed three circular ROIs to measure the mean attenuation (HU) and noise (SD) (***Figure 2***). Three ROIs were placed within the liver right lobe of right portal vein level, abdominal aorta below both renal artery branches, and subcutaneous fat in right buttock. Each ROI was noted to avoid confounding structures, such as large vessels.

### Qualitative Analysis

Two radiologists with 12 and 5 years of experience evaluated each of the five sets, except FBP. For the similar evaluation of the image sets, a coaching session was held for the participating radiologists. Readers were blinded to reconstruction methods and the order of image sets was randomized for each patient. Each reader independently graded the pair-wise approach using a two-monitor high-resolution PACS workstation (EIZO RX 240). The results of one radiologist were used, and those of the other were used to evaluate the inter-reader agreement. Each image set was ranked against one another on a comparative scale for overall image quality, image noise, and image sharpness. A score of 5 was assigned to the images with the best quality. The image sharpness was rated in the evaluation of the liver parenchyma, the pancreas contour, and the kidneys.

### Statistical Analysis

Repeated measures analysis of variance with the Bonferroni post hoc test was used to compare the NPS and TTF of phantom and the HU, and noise in different reconstructions. The Friedman test was used for qualitative analysis. The weighted Cohen’s kappa statistic was used to evaluate agreement. Statistical significance was set at *p* < 0.05. Statistical analyses were performed with SPSS software version 21.0 (IBM Corp.).

## Results

### Phantom Studies

The CTDI_vol_ (mGy) was 2.1, 4.2, 6.3, 8.4, and 10.5. The NPS peak decreased in the order of DLIR-L, M, H. Overall, the NPS peak of DLIR was smaller than that of AV30 or AV50 (***Table 2***).

**Table 2:** Peaks, average spatial frequencies, area under NPS curve in all reconstructions and doses.


	NPS PEAK (HU^2^MM^2^)						

CTDI_VOL_(mGy)	FBP	AV30	AV50	AV100	DLIR-L	DLIR-M	DLIR-H

2.1	1.31	0.88	0.73	0.45	0.68	0.48	0.32

4.2	0.75	0.54	0.45	0.29	0.37	0.27	0.2

6.3	0.48	0.36	0.30	0.21	0.24	0.19	0.14

8.4	0.35	0.28	0.24	0.16	0.18	0.14	0.10

10.5	0.31	0.25	0.22	0.16	0.17	0.14	0.11

	**NPS AVERAGE SPATIAL FREQUENCY (MM^–1^)**						

**CTDI_VOL_(mGy)**	**FBP**	**AV30**	**AV50**	**AV100**	**DLIR-L**	**DLIR-M**	**DLIR-H**

2.1	0.38	0.31	0.27	0.18	0.34	0.33	0.31

4.2	0.36	0.32	0.29	0.19	0.35	0.34	0.33

6.3	0.37	0.33	0.29	0.19	0.35	0.34	0.32

8.4	0.36	0.32	0.29	0.19	0.35	0.34	0.33

10.5	0.36	0.34	0.31	0.20	0.37	0.36	0.34

	**NPS AUC**						

**CTDI_VOL_(mGy)**	**FBP**	**AV30**	**AV50**	**AV100**	**DLIR-L**	**DLIR-M**	**DLIR-H**

2.1	178.9	114.8	80.5	27.2	91.8	66	42.7

4.2	99.5	64.8	46.2	17.7	49.2	35.9	24

6.3	66.7	43.2	31	12.4	33.1	24.5	16.6

8.4	49.6	32.5	23.4	9.4	24.1	17.6	11.7

10.5	42	27.9	20.4	8.9	21.6	16.2	11.2


FBP, filtered back projection; AV30, and AV50 = ASIR-V with a blending factor of 30% and 50%, respectively; DLIR-L, DLIR-M, and DLIR-H, a deep learning-based image reconstruction with low, medium, or high levels, respectively; NPS, noise power spectrum; AUC, area under the curve.

**Figure 3:**
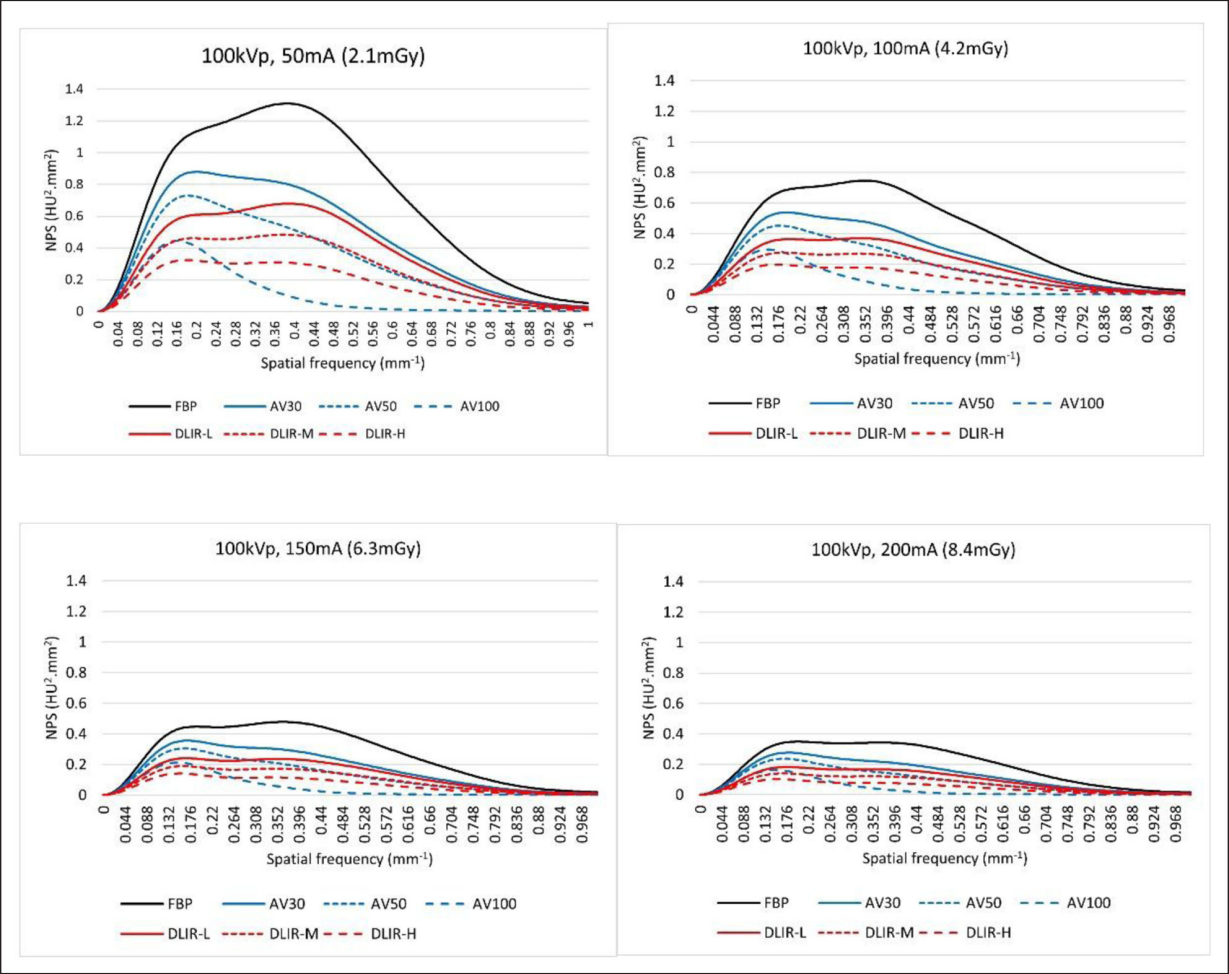
NPS results at different doses and image reconstruction methods: peak kilovoltage (kVp), 100; tube current modulation range 50 mAs **(a)**, 100 mAs **(b)**, 150 mAs **(c)**, and 200mAs **(d)**. FBP, filtered back projection; AV30, and AV50 = ASIR-V with a blending factor of 30% and 50%, respectively; DLIR-L, DLIR-M, and DLIR-H, a deep learning-based image reconstruction with low, medium, or high levels, respectively; NPS, noise power spectrum.

The highest values of the NPS average spatial frequency were obtained for FBP. The NPS spatial frequency decreased as the percentage of AV factor increased and decreased as the DLIR level increased (***Figure 3***). Compared with AV30, the NPS spatial frequencies were 5 to 10% higher with DLIR-L or DLIR-M. Compared with AV50, the NPS spatial frequencies were 10 to 20% higher for all DLIR levels.

For lower-contrast objects, TTF values in images with DLIR were higher than those with AV (***Table 3***). The differences in TTF were greater at low doses. For higher-contrast objects, TTF values did not show significant differences between images with DLIR and those with AV.

**Table 3:** TTF-50s (mm-1) of the 25% ACR phantom CT according to different discs (bone; 955 HU, acrylic; 120 HU) and reconstructions.


CTDI_VOL_	TTF_50_ (MM^–1^) of ROI1 (BONE)							TTF_50_ (MM^–1^) of ROI2 (ACRYLIC)						
	
(mGy)	FBP	AV30	AV50	AV100	DLIR-L	DLIR-M	DLIR-H	FBP	AV30	AV50	AV100	DLIR-L	DLIR-M	DLIR-H

2.1	0.45	0.35	0.45	0.44	0.45	0.44	0.44	0.36	0.35	0.35	0.28	0.40	0.40	0.40

4.2	0.44	0.44	0.44	0.45	0.44	0.44	0.44	0.42	0.41	0.41	0.39	0.44	0.43	0.44

6.3	0.44	0.44	0.44	0.45	0.44	0.44	0.44	0.36	0.35	0.35	0.36	0.37	0.38	0.41

8.4	0.44	0.44	0.44	0.45	0.44	0.44	0.44	0.40	0.39	0.42	0.39	0.44	0.42	0.43

10.5	0.45	0.45	0.45	0.45	0.45	0.44	0.44	0.41	0.42	0.41	0.38	0.44	0.44	0.42


TTF, task-based transfer function; ACR, American College of Radiology; FBP, filtered back projection; AV30, and AV50 = ASIR-V with blending factors of 30%, and 50%, respectively; DLIR-L, DLIR-M, and DLIR-H, a deep learning-based image reconstruction with low, medium, or high levels, respectively.

### Patient Studies

The mean HU showed no significant difference between the six different reconstructions. The SD of the liver and aorta showed significant differences (*p < 0.001*) (***Table 4***). The SD of fat showed significant differences in different protocols, except between AV50 and DLIR-L (*p < 0.001*). A higher factor in AV (AV30 < AV50) and higher strength in DLIR (DLIR-L<DLIR-M<DLIR-H) showed significantly lower SD. Comparison of DLIR images with AV images showed that the SD in DLIR-H and DLIR-M was 10 to 50 % lower than both AV30 and AV50 (*p < 0.001*).

**Table 4:** Mean image noise (HU) according to the image reconstruction method.


RECONSTRUCTION	FBP	AV30	AV50	DLIR-L	DLIR-M	DLIR-H	*P-VALUE*

Liver							

HU	130.46 ± 22.91	130.46 ± 22.91	130.47 ± 22.91	130.63 ± 22.85	130.74 ± 22.86	130.76 ± 22.86	1.000

SD	25.65 ± 1.81	20.03 ± 1.51	16.36 ± 1.34	18.43 ± 1.56	14.40 ± 1.26	10.05 ± 1.00 ^a^	<.001

Aorta							

HU	206.21 ± 50.56	206.47 ± 50.08	206.43 ± 50.07	208.01 ± 50.11	208.11 ± 50.07	206.47 ± 50.08	1.000

SD	27.01 ± 2.51	20.72 ± 2.10	16.69 ± 1.91	19.41 ± 1.97	15.13 ± 1.52	10.50 ± 1.30	<.001

Fat							

HU	107.59 ± 17.71	107.51 ± 17.73	107.49 ± 17.71	106.06 ± 19.58	106.79 ± 17.55	106.58 ± 17.52	1.000

SD	22.56 ± 2.10	17.88 ± 1.77	14.88 ± 1.64	14.82 ± 1.54	11.31 ± 1.32	7.56 ± 1.18	<.001


Data are presented as mean ± standard deviation. The subscripts represent the same group of post hoc analysis (alphabetical order indicates the order, starting from the lowest mean value). P-values were calculated using repeated-measures ANOVA among the six groups. FBP, filtered back projection; AV30, ASIR-V with a blending factor of 30%; AV50, ASIR-V with a blending factor of 50%; DLIR-L, DLIR-M, and DLIR-H, deep learning-based image reconstruction images with low, medium, or high strength levels, respectively; HU, Hounsfield unit; SD, standard deviation.

### Qualitative Analysis

Five reconstruction protocols showed significant differences (*p < 0.001*). The overall image quality was the best for the DLIR-M (*p < 0.001*) (***Table 5***). DLIR-H had the best-ranking score for noise; it provided worse image sharpness compared to DLIR-M and DLIR-L (*p < 0.001*). AV30 and AV50 had relatively lower ranking scores for all aspects compared to the DLIR (*p < 0.001*). Inter-reader agreement was moderate in overall image quality, very good in noise (*K* = 0.48, 0.92, *p < 0.001*) and fair in image sharpness (*K* = 0.24, *p < 0.001*).

**Table 5:** Image quality assessment ranking of the image reconstruction methods.


RECONSTRUCTION	AV30	AV50	DLIR-L	DLIR-M	DLIR-H

Overall image quality	1.93 ± 1.1	1.63 ± 0.78	4.04 ± 0.76	4.51 ± 0.75	2.89 ± 0.84

Noise	1.18 ± 0.39	1.83 ± 0.40	2.99 ± 0.09	4.00 ± 0.00	5.00 ± 0.00

Spatial resolution	2.18 ± 0.67	1.27 ± 0.72	4.67 ± 0.57	4.19 ± 0.60	2.69 ± 0.63±


Data are mean ranking score ± standard deviation. FBP, filtered back projection; AV30, ASIR-V with a blending factor of 30%; AV50, ASIR-V with a blending factor of 50%; DLIR-L, DLIR-M, and DLIR-H, a deep learning-based image reconstruction image with low, medium, or high strength levels.

## Discussion

Our study demonstrated that CT reconstructed with DLIR showed lower noise magnitude and noise texture and image sharpness similar to those with FBP using a phantom and abdominal CT comparing those with AV30 or AV50.

The DLIR was designed to differentiate the signal from noise without changing its texture [18]. In the phantom study, DLIR images with any level showed decreased noise magnitude compared with images with AV30 or 50, which are commonly used in clinical settings for abdominal CT. According to NPS spatial frequency, images with all DLIR levels showed better texture, similar to those with FBP, compared with those of AV50 or AV100. Moreover, images DLIR-L or M showed better texture with those of AV30 and DLIR-H results comparable to those of AV30.

For lower-contrast objects, images with DLIR showed better image sharpness than those with AV. For higher-contrast objects, there were no significant differences between the AV and DLIR images. Previous studies reported that the image sharpness between DLIR and AV50, AV100 was greater for low-contrast objects; however, it also showed differences for high-contrast objects [19]. As our study did not include extremely low doses, different results were obtained.

In the patient study, the measurement of noise with DLIR-M or DLIR-H had lower noise than that with AV30, AV50. CT with DLIR-L did not show significantly different noise compared to AV50. These results were different from those of our phantom study, which showed significantly lower noise in the DLIR-L images.

In the qualitative analysis, DLIR effectively eliminated noise. Jenson et al. showed that readers evaluated images with DLIR-H as the best overall image quality [14]. The authors performed CT with a noise index of 10 [14]. In this study, we performed CT with the noise index of 17. CT with DLIR-M showed the best overall image quality, although DLIR-H showed lower noise. This could be due to image sharpness and texture characteristics. In the phantom study, compared with AV30, NPS spatial frequency were higher with DLIR-L and DLIR-M. It did not show statistically significant differences with DLIR-H. In patient studies, the evaluation of spatial resolution showed a fair inter-reader agreement. Further research is needed on this. The time required for reconstruction is similar between DLIR and AV. Our study showed that DLIR is sufficient for reconstruction as the first option in daily practice.

The present study had several limitations. First, the phantom we used is not in conditions that are very close to the human body. Acrylic insert is a material with a lower HU than bone, and we thought that it could replace the material between water and bone. Further studies are needed for low-contrast materials. Second, this study did not compare the diagnostic capabilities.

In conclusion, phantom data suggests that DLIR showed improved spatial resolution, FBP-like image texture, and effective noise reduction under a decreased radiation dose. Patient data suggests that DLIR showed effective noise reduction while preserving image quality. DLIR-M showed better rankings in both image quality and image sharpness comparing AV-30 or AV-50 in abdominal CT.
